# Hippocampal transcriptome-guided genetic analysis of correlated episodic memory phenotypes in Alzheimer's disease

**DOI:** 10.3389/fgene.2015.00117

**Published:** 2015-03-26

**Authors:** Jingwen Yan, Sungeun Kim, Kwangsik Nho, Rui Chen, Shannon L. Risacher, Jason H. Moore, Andrew J. Saykin, Li Shen, for the Alzheimer's Disease Neuroimaging Initiative

**Affiliations:** ^1^BioHealth, Indiana University School of Informatics and ComputingIndianapolis, IN, USA; ^2^Center for Neuroimaging, Department of Radiology and Imaging Sciences, Indiana University School of MedicineIndianapolis, IN, USA; ^3^Indiana Alzheimer Disease Center, Indiana University School of MedicineIndianapolis, IN, USA; ^4^Computer Science, Dartmouth CollegeHanover, NH, USA; ^5^Genetics, Community and Family Medicine, Geisel School of Medicine at DartmouthLebanon, NH, USA; ^6^Medical and Molecular Genetics, Indiana University School of MedicineIndianapolis, IN, USA; ^7^Center for Computational Biology and Bioinformatics, Indiana University School of MedicineIndianapolis, IN, USA

**Keywords:** genetic associate study, correlated phenotypes, hippocampus, episodic memory, Alzheimer's disease

## Abstract

As the most common type of dementia, Alzheimer's disease (AD) is a neurodegenerative disorder initially manifested by impaired memory performances. While the diagnosis information indicates a dichotomous status of a patient, memory scores have the potential to capture the continuous nature of the disease progression and may provide more insights into the underlying mechanism. In this work, we performed a targeted genetic study of memory scores on an AD cohort to identify the associations between a set of genes highly expressed in the hippocampal region and seven cognitive scores related to episodic memory. Both main effects and interaction effects of the targeted genetic markers on these correlated memory scores were examined. In addition to well-known AD genetic markers *APOE* and *TOMM40*, our analysis identified a new risk gene *NAV2* through the gene-level main effect analysis. *NAV2* was found to be significantly and consistently associated with all seven episodic memory scores. Genetic interaction analysis also yielded a few promising hits warranting further investigation, especially for the RAVLT list B Score.

## Introduction

Alzheimer's disease (AD) is a neurodegenerative disorder that is initially manifested by impaired memory function. Heritability of AD has been estimated to range from 58 to 79% with the evidence collected from familial aggregation, transmission patterns, and large scale twin studies. The lifetime risk of the first-degree relatives of patients can be twice that of the general population (Gatz et al., 2006; Ertekin-Taner, 2010). Given its high heritability, many genetic studies in AD have been performed, including linkage analysis, candidate gene analysis, and genome-wide association study (GWAS). While apolipoprotein E (*APOE*) has been first found (Saunders et al., 1993) and later frequently replicated in many studies (Shen et al., 2010; Hollingworth et al., 2011; Shi et al., 2012), some other markers have also been reported in recent GWAS studies, such as bridging integrator 1 (*BIN1*) (Hu et al., 2011; Lee et al., 2011), clusterin (*CLU*) (Harold et al., 2009; Lambert et al., 2009; Lee et al., 2011), ATP-binding cassette, sub-family A (*ABC1*), member 7 (*ABCA7*) (Hollingworth et al., 2011), complement component (3b/4b) receptor 1 (*CR1*) (Lambert et al., 2009), phosphatidylinositol binding clathrin assembly protein (*PICALM*) (Harold et al., 2009; Lee et al., 2011), EPH receptor A1 (*EPHA1*) (Hollingworth et al., 2011), CD33 molecule (*CD33*) (Hollingworth et al., 2011), Membrane-Spanning 4-Domains (*MS4A4A*/*MS4A6A*), CD2-associated protein (*CD2AP*) (Naj et al., 2011), and 11 new suspectibility loci recently identified in Lambert et al. (2013).

While traditional genetic association studies mostly focus on analyzing the case control status as the phenotype, they are not designed for revealing genetic risk factors associated with relevant quantitative phenotypes where hidden signals may have appeared long before the disease diagnosis is confirmed. Cognitive measures have been used as highly relevant quantitative traits (QTs) for neuropsychiatric conditions (Gottesman and Gould, 2003; Glahn et al., 2007), including AD (Bennett et al., 2009). These intermediate measures quantitatively capture the progressive nature of the disease and are believed to hold great promise in identifying genetic risk factors. As one of the primary preceding signals, memory decline has been observed in many AD patients prior to diagnostically cognitive, behavioral, and social changes (Backman et al., 2001). Thus, relating genetic markers to memory QTs, rather than diagnosis, has great potential to improve the mechanistic understanding of the pathway from genetics to cognition and then to diagnosis.

Accompanied with advances in high-throughput genotyping techniques, substantial efforts have recently been made to facilitate the reliable identification of memory-related genes. Several genes, including previously identified AD risk genes, have been reported to contribute to the episodic memory disturbances, including *APOE*, *CLU*, *BIN1*, brain-derived neurotrophic factor (*BDNF*), and WW and C2 domain containing 1 (*WWC1*/*KIBRA*) (Egan et al., 2003; Burgess et al., 2011). Recently, a new risk gene, FAST kinase domains 2 (*FASTKD2*), has been reported in the GWAS of a large cohort (Ramanan et al., 2014). However, most of these studies focus only on one specific episodic memory scores. In this study, we aim to identify the genetic factors that are jointly associated with multiple correlated episodic memory scores. This may have the advantage of reducing the biases introduced by variability and outliers in the analysis of a single score, and also to some extent consolidating and integrating the findings.

Existing genetic findings mostly consist of individual single nucleotide polymorphism (SNP) markers or genes; and they typically explain a part of heritability but not all. To search for additional heritability, we extended our analysis to incorporate both single marker tests and pair-wise SNP interaction tests. Given the major combinatorial explosion challenge for SNP-SNP interaction analysis, we performed our analysis in a targeted fashion to make computation feasible. In this work, we only examined the genes that are highly preferred to express in the hippocampal region, which is a critical structure related to learning and memory. We performed an association study to examine the relationships between the SNPs in these genes and seven cognitive scores representing episodic memory, including two scores from Logical Memory Test, and five scores from the Rey Auditory Verbal Learning Test (RAVLT). We performed multiple comparison correction, by considering both the correlation structure within the genotyping data and that among memory scores.

## Materials and methods

### Alzheimer's disease imaging initiative (ADNI)

Genotype and QT data used in this study were obtained from the Alzheimer's Disease Neuroimaging Initiative (ADNI) database (adni.loni.usc.edu). The ADNI was launched in 2003 by the National Institute on Aging (NIA), the National Institute of Biomedical Imaging and Bioengineering (NIBIB), the Food and Drug Administration (FDA), private pharmaceutical companies and non-profit organizations, as a $60 million, 5-year public- private partnership. The primary goal of ADNI has been to test whether serial magnetic resonance imaging (MRI), positron emission tomography (PET), other biological markers, and clinical and neuropsychological assessment can be combined to measure the progression of mild cognitive impairment (MCI) and early AD. Determination of sensitive and specific markers of very early AD progression is critical to aid researchers and clinicians to develop new treatments and monitor their effectiveness, and to reduce the time and cost of clinical trials.

The Principal Investigator of this initiative is Michael W. Weiner, MD, VA Medical Center and University of California, San Francisco. ADNI is the result of efforts of many co-investigators from a broad range of academic institutions and private corporations, and subjects have been recruited from over 50 sites across the U.S. and Canada. The initial goal of ADNI was to recruit 800 subjects but ADNI has been followed by ADNI-GO and ADNI-2. To date these three protocols have recruited over 1500 adults, ages 55–90, to participate in the research, consisting of cognitively normal older individuals, people with early or late MCI (EMCI or LMCI), and people with early AD. The follow up duration of each group is specified in the protocols for ADNI-1, ADNI-2, and ADNI-GO. Subjects originally recruited for ADNI-1 and ADNI-GO had the option to be followed in ADNI-2. Thousands of longitudinal imaging scans (Jack et al., 2008; Jagust et al., 2010), performance on neuropsychological and clinical assessments (Petersen et al., 2010) and biological samples (Shaw et al., 2009) were collected at baseline and at follow-up visits for all or a subset of participants. Genome-wide genotyping data (Saykin et al., 2010) are available on the full ADNI sample. For up-to-date information, see www.adni-info.org.

### Subjects

To eliminate the possible bias introduced by population stratification, this study was restricted to non-Hispanic Caucasian participants from both the ADNI-1 and ADNI-2/GO cohorts. Subjects in other racial/ethnic groups were excluded in the analysis due to the relative small number of those samples (less than 10%). We employed the population stratification approach used in Kim et al. (2013). Briefly, 988 founders with known ancestry information from HapMap phase 3 (HapMap3) release 2 were used as reference data in the population stratification step and merged with the ADNI samples. Multidimensional scaling (MDS) was performed using PLINK with identity-by-state (IBS) pairwise distance matrix of the merged data. We compared all ADNI participants with self-reported race/ethnicity as “non-Hispanic/white” with HapMap3 samples in the MDS space, and excluded those participants grouped with HapMap3 samples whose ancestries were neither CEU nor TSI. As a result, 1149 non-Hispanic Caucasian participants were included in this study, and their GWAS data passed the above population stratification and all the other quality control (QC) procedures described in Kim et al. (2013). Shown in Table 1 is the demographic information for these subjects.

**Table 1:** **Demographics of participants**.

	**HC**	**eMCI**	**LMCI**	**AD**
Number	325	191	427	206
Gender(M/F)	173/152	106/85	274/153	115/91
Handedness(R/L)	302/23	170/21	385/42	189/17
Age(mean ± std)	75.36±5.32	70.80±7.37	74.49±7.47	75.50±7.98
Education(mean ± std)	16.27±2.67	15.91±2.66	15.86±2.94	14.95±3.07
Log_Delay (mean ± std)	13.35±3.34	8.88±1.71	3.87±2.69	1.34±1.95
Log_IMM (mean ± std)	14.14±3.28	10.94±2.83	7.14±3.14	4.10±2.86
RAV_Recog (mean ± std)	12.89±2.40	12.01±2.70	9.63±3.65	7.19±3.94
RAV_T6 (mean ± std)	8.22±3.53	7.07±3.76	3.87±3.22	1.70±1.84
RAV_T30 (mean ± std)	7.29±3.78	5.84±4.09	2.83±3.31	0.73±1.56
RAV_TB (mean ± std)	5.01±1.87	4.51±1.96	3.65±1.54	2.89±1.31
RAV_TOTAL (mean ± std)	43.69±s 9.94	39.41±10.70	31.04±9.32	22.63±8.16

### Candidate gene selection

**Figure 1:**
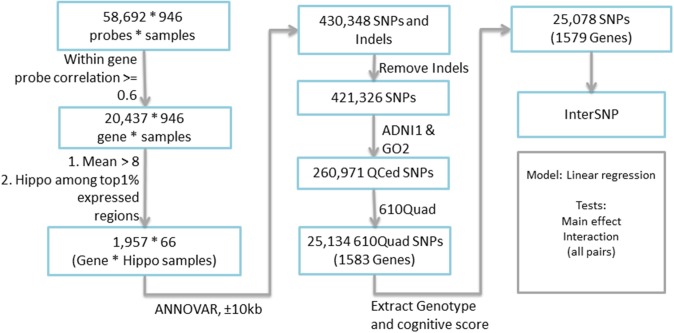
**Complete workflow for candidate gene selection**.

Candidate genes were extracted based on the expression profile obtained from the Allen Human Brain Atlas (AHBA) (www.brain-map.org). AHBA provides a comprehensive expression mapping of ~60,000 probes (~30,000 genes and transcripts) across the human brain, with ~1000 brain samples collected for two full brains and ~500 brain samples for the other six half brains. In a recent study, extremely high similarity (95%) among the expression profiles of these brain samples was reported (Zeng et al., 2012), and therefore this study employed only one full brain to identify the candidate genes. Expression data of all probes, targeting 29,196 genes and transcripts, across the whole brain were downloaded. Since the expression data was measured based on the probes and each gene has several probes, we excluded those genes whose multiple probe expression profiles do not correlate very well (i.e., correlation coefficient <0.6). The final expression level of each gene was measured based on the average of highly correlated probes, while other probes were considered as outliers. Each brain sample location in AHBA was mapped back to the MarsBaR AAL atlas with 106 brain ROIs (Tzourio-Mazoyer et al., 2002). We calculated the ROI level expression of each gene by averaging the expression of all brain samples within each ROI. Totally 66 out of 948 brain samples were located within the hippocampal region defined by the MarsBaR AAL atlas. Ultimately 1957 genes with average expression level >8 and the hippocampus as one of its top 1% expressed regions were extracted for further analysis. The overall workflow is shown in Figure 1.

### Genotype and memory data

Genotype data of all non-Hispanic Caucasian participants from both ADNI-1 and ADNI-GO/2 were downloaded, quality controlled, imputed to the Illumina 610 Quad platform and combined. As mentioned earlier, population stratification was performed using the approach described in Kim et al. (2013) to make sure that all the participants were non-Hispanic Caucasian. Among all 1957 selected candidate genes, 25,134 SNPs from 1583 genes (boundary: ±10 kb) were found based on ANNOVAR (http://www.openbioinformatics.org/annovar/). Fifty six SNPs from four genes with missing genotype data were excluded. Totally our analysis included 1579 genes and 25,078 SNPs. Seven episodic memory related cognitive scores (Table 2) were downloaded as memory QTs, including (1) immediate recall and delayed recall scores from Logical Memory Test, and (2) trials I–V, List B, immediate recall, 30-min delayed recall, and recognition from Rey Auditory Verbal Learning Test (RAVLT). Final analysis was performed using INTERSNP (Herold et al., 2009) to examine both main and interaction effects. Pairwise interactions were evaluated among all 25,078 SNPs.

**Table 2:** **Description of seven episodic memory scores**.

**Cognitive Score**	**Description**
Log_IMM	Screening logical memory immediate recall
Log_Delay	Screening logical memory delayed recall
RAV_TOT	Baseline RAVLT total score
RAV_TOT6	Baseline RAVLT—trial 6 total number of words recalled
RAV_TOTB	Baseline RAVLT—List B total number of words recalled
RAV_T30	Baseline RAVLT—30 min delay total
RAV_Recog	Baseline RAVLT—30 min delay recognition score

### Statistical analysis

This work focuses on the analysis of the genes that are highly preferred to express in the hippocampal region. One of the goals is to investigate both main and epistasis effects of these genes/SNPs on the memory performance measured by seven correlated cognitive scores. Traditional linear regression was first performed to test the main effect of each SNP. A full genetic interaction model was then applied on each pair of SNPs. Potential confounding factors, including baseline age, gender, education, and handedness, were incorporated as covariates in both analyses to exclude their effects.

The whole procedure was performed using the INTERSNP software (Herold et al., 2009). It was designed specifically for genome wide interaction analysis of relating SNPs to the case control conditions or various QTs. In this study, with memory scores as QTs, two linear regression models were used to capture the influence of each interaction pair. The first model took into account the main effects of both SNPs plus all the covariates mentioned above, while the second model had an extra interaction term. Comparison between these two models yielded the final interaction effect. Detailed information can be found in http://intersnp.meb.uni-bonn.de/manual.html.

#### Correction for multiple testing

Due to the existence of the linkage disequilibrium (LD) structures within SNPs and high correlation among episodic memory measures (Table 3), direct application of either Bonferroni or Benjamin Hochberg correction would be overly conservative and may screen out a lot of potential signals with high false negative rates. In this study, we first estimated the LD blocks in PLINK using an independent data set from the 1000 Genomes Project, and employed the estimated LD block number (*N_LD_* = 5004) as the independent genetic test number. Similarly for seven cognitive test scores, we estimated the number of independent QTs based on eigenvalues (*N_iQT_* = 2) by applying the matrix spectral decomposition, using the method described in Van Der Sluis et al. (2013), Pedraza et al. (2014). In the analysis of main effects, using SNP-level *p*-values as the input, we performed a gene-based integration based on VEGAS (Liu et al., 2010), where gene level significance was obtained through one million permutations and further corrected for the gene number (*N_G_* = 1579) and the estimated independent QT number (*N_iQT_* = 2) using the Bonferroni procedure (i.e., corrected *p*-value = uncorrected *p*-value × (*N_G_* × *N_iQT_*)). In the analysis of interaction effects, multiple comparison correction was also performed using the Bonferroni procedure based on *N_LD_* (*N_LD_* −1)/2 = 5004 × 5003/2 = 12, 517, 506 unique LD block pairs and the estimated independent QT number *N_iQT_* = 2 [i.e., corrected *p*-value = uncorrected *p*-value × (12, 517, 506 × 2)].

**Table 3:**
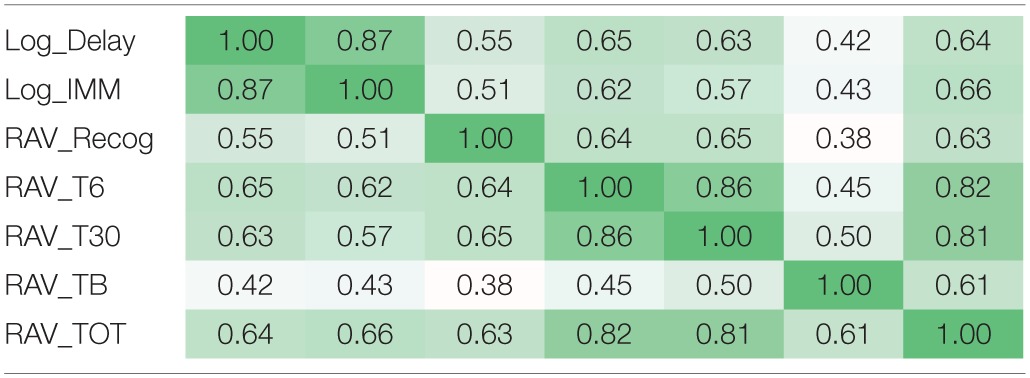
**Correlation structure among seven memory scores**.

## Results

### Main effects

**Figure 2:**
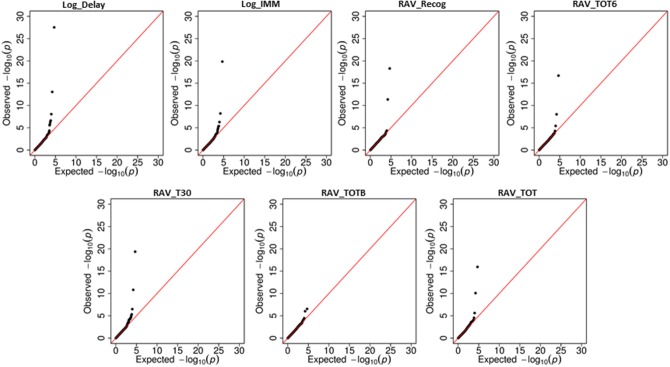
**Quantile-Quantile (Q-Q) plots for the main effect analyses of seven cognitive scores**.

Linear regression was first used to regress 25,078 SNPs on seven cognitive scores, respectively. SNP level *p*-values obtained from INTERSNP (see top SNPs in Supplemental Table S1) was further analyzed through VEGAS to obtain gene level *p*-values. Reference genome in VEGAS was changed to hg19 to be consistent with our data set. Shown in Figure 2 are the Q-Q plots for the genetic main effect analyses of seven cognitive scores. In total, eight genes from six different chromosomes have been identified with corrected *p*-value ≤ 0.05 [i.e., uncorrected gene-level *p*-values ≤ 0.05/(*N_G_* × *N_iQT_*) = 1.6E-05]. Shown in Table 4 are the gene findings along with *p*-values for seven cognitive scores. As observed, the well-known AD risk gene *APOE* is associated with six cognitive scores. Two genes are consistently associated with all seven memory scores: neuron navigator 2 (*NAV2*) and Translocase Of Outer Mitochondrial Membrane 40 Homolog (*TOMM40*), where *TOMM40* is proximal to *APOE* and has been reported to be associated with the memory impairment (Berbee et al., 2011). Four other genes, Protein Kinase, AMP-Activated, Gamma 2 Non-Catalytic Subunit (*PRKAG2*), protein tyrosine phosphatase, receptor type, D (*PTPRD*), CUGBP, elav-like family member 2 (*CELF2*), and PDS5, regulator of cohesion maintenance, homolog B (*PDS5B*) are each significantly associated with two cognitive scores. Protocadherin 9 (*PCDH9*) is significantly associated with RAVLT list B scores.

**Table 4:** **Gene-level main effects identified in seven memory scores**.

**Chr**		**7**	**9**	**10**	**11**	**13**	**13**	**19**	**19**
**Gene**		**PRKAG2**	**PTPRD**	**CELF2**	**NAV2**	**PCDH9**	**PDS5B**	**APOE**	**TOMM40**
Log_Delay	orig_p	2.5E-03	2.5E-05	4.7E-04	**<1.0E-06**	5.0E-04	**3.0E-06**	**<1.0E-06**	**<1.0E-06**
	corr_p	NS	NS	NS	**<3.2E-03**	NS	**9.5E-03**	**<3.2E-03**	**<3.2E-03**
Log_IMM	orig_p	3.2E-04	1.0E-04	2.8E-05	**<1.0E-06**	4.5E-03	**1.3E-05**	**<1.0E-06**	**<1.0E-06**
	corr_p	NS	NS	NS	**<3.2E-03**	NS	**4.1E-02**	**<3.2E-03**	**<3.2E-03**
RAV_Recog	orig_p	9.3E-03	2.1E-03	3.2E-05	**1.0E-06**	1.7E-03	5.8E-02	**<1.0E-06**	**<1.0E-06**
	corr_p	NS	NS	NS	**3.2E-03**	NS	NS	**<3.2E-03**	**<3.2E-03**
RAV_T6	orig_p	**4.0E-06**	**8.0E-06**	1.1E-04	**<1.0E-06**	2.0E-02	4.2E-04	**<1.0E-06**	**<1.0E-06**
	corr_p	**1.3E-02**	**2.5E-02**	NS	**<3.2E-03**	NS	NS	**<3.2E-03**	**<3.2E-03**
RAV_T30	orig_p	4.8E-04	**3.0E-06**	**1.5E-05**	**<1.0E-06**	1.3E-03	2.4E-03	**<1.0E-06**	**<1.0E-06**
	corr_p	NS	**9.5E-03**	**4.7E-02**	**<3.2E-03**	NS	NS	**<3.2E-03**	**<3.2E-03**
RAV_TB	orig_p	5.4E-02	5.1E-05	1.8E-03	**2.0E-06**	**<1.0E-06**	4.3E-04	1.2E-04	**<1.0E-06**
	corr_p	NS	NS	NS	**6.3E-03**	**<3.2E-03**	NS	NS	**<3.2E-03**
RAV_TOT	orig_p	**1.4E-05**	2.0E-04	**<1.0E-06**	**<1.0E-06**	1.5E-03	3.9E-04	**<1.0E-06**	**<1.0E-06**
	corr_p	**4.4E-02**	NS	**<3.2E-03**	**<3.2E-03**	NS	NS	**<3.2E-03**	**<3.2E-03**

*Both original and corrected p-values are shown. NS indicates “not significant” (i.e., corrected p > 0.05). Bolded records indicate significant associations with corrected p ≤ 0.05*.

### Interaction effects

Similarly, interaction analysis was also performed based on the linear regression model in INTERSNP, where the association between each pair of 25,078 candidate SNPs and each of seven cognitive scores was examined. After Bonferroni correction using the estimated independent test number, no significant interaction was observed for Log_Delay, RAV_TOT6, and RAV_T30. Two interactions between gene *PTPRD* (rs598356 and rs610789, in the same LD block) and gene *KHSRP* (rs2075755) were found to pass the significance threshold of corrected *p* = 0.05 (or uncorrected *p* = 2.0 × 10^−9^) for Log_IMM. Three interactions, between *FLJ39653* and *SOX5*, *FBXO45* and *SOX5*, *FHIT*, and *PRB1*, were found for RAV_Recog. Only one interaction, between *RCC2* and *ZDHHC21*, passed the significance threshold in RAV_TOT test. A large number of interactions were observed to affect RAV_TOTB, which was the list B recall score in RAVLT test. In total, 71 interactions were identified among 46 genes with corrected *p* = 0.05. Shown in Table 5 is the list of interactions with uncorrected *p* ≤ 1 × 10^−8^, where bolded records indicate significant interactions with corrected *p* ≤ 0.05. The list of interaction findings for RAV_TOTB is shown in Supplemental Table S2, where 298 interactions have uncorrected *p* = 1 × 10^−8^, and 71 have corrected *p* = 0.05.

**Table 5:** **Interaction SNP pairs identified in Log_Delay, Log_IMM, RAV_Recog, RAV_TOT6, and RAV_TOT with uncorrected *p* ≤ 1 × 10^−8^**.

**Test**	**Chr_1**	**rs_No_1**	**Gene_1**	**Dist_1**	**Chr_2**	**rs_No_2**	**Gene_2**	**Dist_2**	***p*-value**	**corr_p**
Log_Delay	3	rs10866046	*FHIT*	0	5	rs255211	*MCTP1*	0	3.58E-09	0.090
	9	rs598356	*PTPRD*	0	19	rs2075755	*KHSRP*	0	6.19E-09	0.155
	9	rs610789	*PTPRD*	0	19	rs2075755	*KHSRP*	0	7.35E-09	0.184
Log_IMM	**9**	**rs598356**	***PTPRD***	**0**	**19**	**rs2075755**	***KHSRP***	**0**	**8.74E-10**	**0.022**
	**9**	**rs610789**	***PTPRD***	**0**	**19**	**rs2075755**	***KHSRP***	**0**	**1.16E-09**	**0.029**
	4	rs1980187	*ENOPH1*	0	4	rs17579878	*ANK2*	0	3.24E-09	0.081
	4	rs6827820	*ENOPH1*	6647	4	rs17579878	*ANK2*	0	3.54E-09	0.089
	3	rs213376	*FHIT*	0	6	rs6909677	*SASH1*	0	4.68E-09	0.117
RAV_Recog	**4**	**rs6822469**	***FLJ39653***	**0**	**12**	**rs1464502**	***SOX5***	**0**	**4.77E-10**	**0.012**
	**3**	**rs9843585**	***FBXO45***	**2925**	**12**	**rs16926727**	***SOX5***	**0**	**8.76E-10**	**0.022**
	**3**	**rs17063416**	***FHIT***	**0**	**12**	**rs2059764**	***PRB1***	**–1550**	**1.44E-09**	**0.036**
	3	rs3915504	*FHIT*	0	14	rs17510215	*NRXN3*	0	4.1E-09	0.103
	12	rs1513126	*RASSF8*	0	17	rs8066154	*NUFIP2*	0	4.38E-09	0.110
	2	rs4849056	*ZC3H8*	−6928	9	rs10511506	*PTPRD*	0	7.99E-09	0.200
	7	rs2190107	*GPR37*	0	14	rs2268952	*AKAP6*	0	8.21E-09	0.206
RAV_TOT6	11	rs7118965	*OPCML*	0	16	rs3866638	*WWOX*	0	9.71E-09	0.243
RAV_TOT	**1**	**rs2883272**	***RCC2***	**8100**	**9**	**rs7853156**	***ZDHHC21***	**0**	**1.76E-09**	**0.044**
	6	rs1743448	*KHDRBS2*	0	18	rs10401068	*DOK6*	0	3.45E-09	0.086
	1	rs2883272	*RCC2*	8100	9	rs10961636	*ZDHHC21*	0	3.74E-09	0.094

*No interaction was observed at this threshold for RAV_T30. List of interaction findings for RAV_TB is available in Supplemental Table S2. Bolded records indicate significant interactions with corrected p ≤ 0.05*.

## Discussion

Among all eight genes identified in our main effect analysis, *APOE* is significantly associated with six cognitive scores, while two other genes, *NAV2*, and *TOMM40* are observed to be significantly associated with all seven cognitive scores. Two of them, *APOE* and *TOMM40* have been widely studied and known as AD risk genes. For the new candidate gene *NAV2*, despite no direct association has been previously reported between *NAV2* and AD or episodic memory, its special role in neurite growth and cell migration (Muley et al., 2008; Mcneill et al., 2010; Shioya et al., 2010; Marzinke et al., 2013) suggests that it warrants further investigation as a potential target in future analyses. In addition, the expression of *NAV3*, a paralog of *NAV2*, was reported to be enhanced in degenerating pyramidal neurons in the cerebral cortex of AD (Shioya et al., 2010).

Each of the other five genes (*PRKAG2*, *PTPRD*, *CELF2*, *PDS5B*, and *PCDH9*) is associated with one or two cognitive scores in our study. Most of them have already been previously reported as being associated with cognitive impairment. The *PRKAG2* gene provides instructions for making one part (the gamma-2 subunit) of a larger enzyme called AMP-activated protein kinase (*AMPK*), which is a master switch of energy and plays an important role in metabolism functions. Better performance in verbal memories and attention tasks has been found for specific genotype groups −26 polymorphism *PRKAG2*, indicating an active role of *PRKAG2* in cognitive impairment (Kim et al., 2012). This finding has been confirmed recently in another network based analysis (Caberlotto et al., 2013). Mostly known as tumor suppressor gene (Stallings et al., 2006; Kohno et al., 2010), *PTPRD* was also discovered to have possible functional connections with neurological disorders (Ghani et al., 2012) and may have potential interaction with AD marker tau protein (Shulman et al., 2014). *CELF2* functions to induce the exon 2/3 skipping in *MAPT* gene, which encodes the AD risk protein tau (Ladd, 2013). *PDS5B*, regulator of cohesion maintenance, has been found to be significantly associated with brain atrophy in Furney et al. (2011).

**Figure 3:**
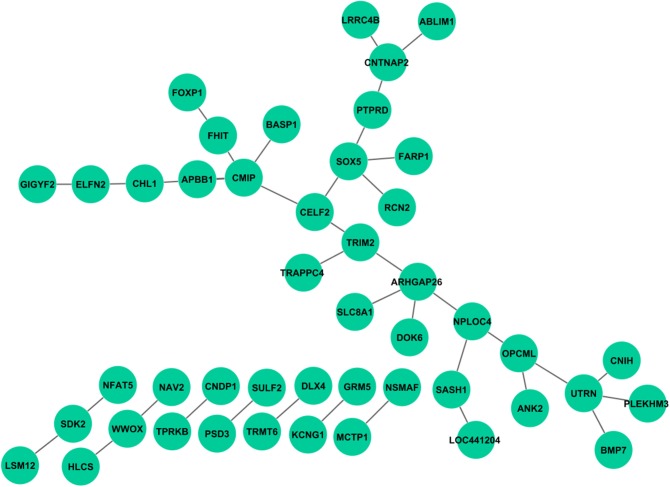
**Genetic interactions associated with RAV_TOTB. Each link indicates at least one pair of SNPs between two genes reaches statistical significance with corrected *p* < 0.05**.

Unlike the main effect tests, no interactions were observed to be associated with all cognitive scores. After Bonferroni correction based on estimated independent genetic and memory QT numbers, no interaction was found for Log_Delay, RAV_TOT6, and RAV_T30. A few interaction signals, ranging from 1 to 3, were found to be associated with Log_IMM, RAV_Recog, or RAV_TOT. Interestingly, a large number of interactions were found in RAV_TOTB. Compared with interactions from the STRING database (Franceschini et al., 2013), no overlap has been found. We mapped SNPs to genes, and plotted the interaction network using Cytoscape (Shannon et al., 2003); see Figure 3. Enrichment analysis was performed using MetaCore from Thomson Routers and GSEA (Mootha et al., 2003; Subramanian et al., 2005), respectively. In MetaCore, we did not find any enriched pathways with FDR *q*-value <0.05. Gene set enrichment analysis in GSEA yielded 77 enriched sets with FDR *q*-value < 0.05, which can generally be categorized into 3 groups: (1) genes with promoter regions containing a specific motif, (2) targets of microRNAs, and (3) genes in cancer module.

In sum, we have performed single SNP/gene analysis and SNP interaction analysis on genes highly expressed in the hippocampal region to identify the genetic factors that are jointly associated with multiple correlated episodic memory scores, in order to reduce the biases introduced by the noise and outliners in the individual analysis of each single score. Three genes were identified to be significantly associated with most of the cognitive scores. *NAV2* is a novel candidate risk gene whereas the other two (*APOE* and *TOMM40*) have been previously reported in AD studies. Although it is not well studied in the AD field, the essential role of *NAV2* in neurite outgrowth and cell migration makes it a potential target warranting further investigation. Unlike the main effect analysis, we did not find any interaction signals that consistently influence all or most of the seven memory scores. Most memory scores have none or few interaction signals observed except RAV_TOTB, which has over 70 interaction pairs passing the significance threshold. Replication and validation of newly identified interactions warrant further investigation.

### Conflict of interest statement

The authors declare that the research was conducted in the absence of any commercial or financial relationships that could be construed as a potential conflict of interest.
